# LncRNA SNHG1 promotes EMT process in gastric cancer cells through regulation of the miR-15b/DCLK1/Notch1 axis

**DOI:** 10.1186/s12876-020-01272-5

**Published:** 2020-05-18

**Authors:** Zhi-Qi Liu, Wei-Feng He, Yang-Jie Wu, Shun-Li Zhao, Ling Wang, Yan-Yi Ouyang, San-Yuan Tang

**Affiliations:** 1 Oncology Department, Brain Hospital of Hunan Province, No.427, Section, 3, Furong Middle Road, Changsha, 410007 Hunan Province People’s Republic of China; 2 Oncology Department of Medical, The First Affiliated hospital, University of South China, Hengyang, 421000 People’s Republic of China; 3 Yichang Central People’s Hospital, Yichang, 443000 People’s Republic of China; 4 Hengyang Central Hospital, Hengyang, 421000 People’s Republic of China

**Keywords:** Gastric cancer, lncRNA SNHG1, DCLK1/Notch1, miR-15b

## Abstract

**Background:**

Gastric cancer (GC) is a malignant tumour originating from the gastric mucosa epithelium that seriously threatens human health. DCLK1, miR-15b and lncRNA SNHG1 play potential roles in the occurrence of GC, but the mechanism remains unclear.

**Methods:**

Gene expression of DCLK1, miR-15b and lncRNA SNHG1 was investigated by qRT-PCR. Protein expression was detected by Western blotting. Migration and invasion of gastric cancer cells was tested by a Transwell assay and wound healing assay. Cell proliferation was measured by an MTT assay. Finally, the correctness of the prediction results was confirmed by a dual-luciferase reporter assay.

**Results:**

The expression of DCLK1, Notch1, and SNHG1 was increased in GC tissues, while the expression of miR-15b was decreased. Overexpression of lncRNA SNHG1 promoted the expression of DCLK1 and Nothc1 in GC cells. Moreover, miR-15b targeted DCLK1 to regulate Notch1 expression and inhibited the EMT process in GC cells. SNHG1 enhanced the effects of DCLK1/Notch1 on the EMT process through regulating miR-15b expression.

**Conclusion:**

SNHG1 enhances the EMT process in GC cells through DCLK1-mediated Notch1 pathway, which can be a potential target for treating GC.

## Background

Gastric cancer (GC) is a type of malignant tumour with a very high incidence. The poor prognosis and survival of GC are due to it being diagnosed in an advanced stage and the limited response to tumour metastasis [1]. The epithelial-mesenchymal transition (EMT) process is an important cell behaviour regulating tumour cell migration; thus, it is of great significance to explore the impact of EMT on gastric cancer [2].

Doublecortin-like kinase 1 (DCLK1) is a transmembrane microtubule-related kinase which results in nervous system development [3]. In addition, it includes a doublecortin domain which modulates a C-terminal serine-threonine protein kinase domain [4]. DCLK1 has been reported to be closely related to the occurrence and metastasis of rectal cancer [5]. Moreover, the prognosis of GC patients was worse with the increase in DCLK1 expression in tissues of patients [6], and DCLK1 can activate Notch signalling pathway [7]. Although there is great progress in the study of the relationship between DCLK1 and cancer, the impact of DCLK1 on the EMT process of gastric cancer remains unclear.

LncRNA small nucleolar RNA host gene 1 (SNHG1) is a long-chain, non-coding RNA associated with EMT in cancer cells [8–10]. It has been reported that lncRNA SNHG1 can enhance the growth and progression of cervical cancer cells [11]. Additionally, previous studies have shown that the differential expression of lncRNA SNHG1 is upregulated in gastric cancer [12], indicating that it is associated with the development of gastric cancer. Meanwhile, miR-15b belongs to the microRNA family, and it is a non-coding RNA that directly regulates protein expression. It has been previously confirmed that miR-15b plays a crucial role during the metastasis of colorectal cancer [13], whereas its expression is low in GC [14]. Meanwhile, software has been used to predict that miR-15b is one of the targets of lncRNA SNHG1. However, the mechanism of either lncRNA SNHG1 or miR-15b in GC remains to be studied. As the Notch1 signalling pathway could be suppressed by silencing SNHG1 in laryngeal cancer [15], our study aimed to explore the role of Notch1 in GC development.

Based on this background, we supposed that lncRNA SNHG1 promotes EMT in gastric cancer cells via the miR-15b-regulated DCLK1/Notch1 axis. Therefore, a series of experiments was used to verify whether DCLK1, Notch, and SNHG1 are highly expressed in GC cell lines, and we aimed to determine the role of DCLK1, miR15b and lncRNA SNHG1 in GC to find new strategies for treating GC.

## Methods

### Cell lines and cell culture

Human gastric mucosal cell line (GES-1 cells) and human gastric cancer cell lines (N87, SGC7901 and MKN-28 cells) were purchased from the American Type Culture Collection (ATCC, Rockville, MD, USA). The cells were cultured at 37 °C with 5% CO_2_ in PRMI-1640 medium (Gibco, USA) containing 10% foetal bovine serum (Thermo Fisher, USA). BR-V-108 plasmids vector and TOP10 *E. coli* competent cells (TIANGEN, China) were used.

### Lentiviral vector preparation

Target gene RNA interference CCACAGGACAATGCTGAACTT lentiviral vector (Shanghai Biosciences, Co., Lt., Shanghai, China) was established by designing RNA interference target sequences using the DCLK1 gene used as a template. A 50 μl reaction system was prepared according to the NEB instructions, and the BR-V-108 vector was double-digested with AgeI and EcoRI to linearize it, transferring the ligation product into prepared TOP10 *E. coli* competent cells. PCR was used to identify positive recombinants, and sequencing was performed. The sequencing results were compared with the correct clones for plasmid extraction.

### qRT-PCR

Total RNA isolated from tissues or cells was monitored with Trizol reagent (Invitrogen, USA) according to the manufacturer’s instruction, and the cDNA Synthesis system was used to determine reverse transcription. The samples were run using the following cycling parameters: 95 °C 10 s, 1 cycle and 95 °C 5 s, 60 °C 30 s, 45 cycles. The relative expression of RNA was calculated with the 2^-∆∆CT^ method using GAPDH as an internal control.

### Western blot

Whole-cell lysates were collected using RIPA buffer. Proteins were separated using 10% SDS polyacrylamide gel, and the gels were transported to PVDF membranes (Thermo Fisher Scientific, CA, USA). The PVDF membranes were incubated with 5% skim milk in TBST at room temperature for 1 h. Later on, the PVDF membranes were probed with primary antibodies: anti-DCLK1 antibody (1:1000, Abcam, CA, USA), anti-Notch1 antibody (1:3000, Abcam, CA, USA), anti-E-cadherin (1:3000, Abcam, CA, USA), anti-Vimentin (1:3000, Abcam, CA, USA), anti-slug (1:1000, Abcam, CA, USA), anti-TGF-β (1:1000, Abcam, CA, USA), anti-MMP2 (1:1000, Abcam, CA, USA), anti-MMP9 (1:1000, Abcam, CA, USA) and anti-GAPDH antibody (1:3000, Bioworld, CA, USA) overnight at 4 °C. After that, the PVDF membrane was incubated for 1 h in secondary antibody anti-rabbit IgG second antibody (Abcam; ab150077) (1:5000) at room temperature for 1 h. Finally, the immunoreactivity was detected using ECL reagent (Santa Cruz Biotechnology).

### Dual-luciferase reporter assay

3’UTR of SNHG1 or DCLK1 wild-type mutant fragments were cloned into pmirGLO Dual-Luciferase miRNA Target Expression Vector (Promega, USA). MiR-15b mimics or inhibitors and the recombinant vector were co-transfected by lipofectamine 3000 in the cells. The transfection and harvest efficiencies were controlled for using the pmirGLO reporter as an internal control. The chemiluminescence of luciferase activity was measured using a dual-luciferase reporter assay (Promega, USA) according to the manufacturer’s protocol.

### MTT assay

The 5-diphenyltetrazolium bromide (MTT) assay was performed to examine the proliferation of the gastric cancer cells. In brief, cells were seeded on 96-well plates (5 × 10^3^/well) and incubated at 37 °C for 24, 48 and 72 h. Then, the cells were incubated with 100 μl 0.5 mg/ml MTT for another 4 h at 37 °C and dissolved in 150 μl dimethylsulfoxide (DMSO) per well. Finally, the optical density value of each well at 570 nm was examined by a microplate reader (Thermo Fisher Scientific).

### Wound healing assay

The GC cells were transfected for 48 h and isolated to make a final concentration at 2 × 10^5^ ml^− 1^, and they were then plated in 12-well plates (2 × 10^5^ per well) for 24 h. When the cells reached 100% confluence, sterile pipette tips were used to scratch the wound uniformly. Cell motility was assessed by measuring the movement of cells into a scraped wound. The speed of wound closure was monitored after 24 h by measuring the distance of the wound from 0 h. Each experiment was conducted in triplicate.

### Transwell invasion experiment

For the cell invasion analysis, a Transwell assay was performed in this study. The upper chamber was pre-treated with 100 μl of Matrigel. GC cells were seeded into the upper chamber in media with 1% FBS, and the density was adjusted to approximately 1.0 × 10^4^ cells per chamber. RPMI1640 medium with 10% FBS was added in the lower chamber. After 24 h of incubation at 37 °C, the Transwell chamber was rinsed twice with PBS (5 min per time), fixed by 5% glutaraldehyde at 4 °C and stained with 0.1% crystal violet for 30 min. The Transwell chamber was washed twice with PBS and then observed under a microscope. The number of cells invading the Matrigel was regarded to be a reflection of the invasion ability.

### Statistical analysis

At least three independent experiments were performed in each group, and all values were expressed as the mean ± standard deviation (SD). The comparison between two groups was analysed by Student’s t-test. The comparisons among multiple groups were made with a one-way analysis of variance (ANOVA) followed by Tukey’s test (GraphPad Prism7). *P* < 0.05 was considered to indicate a significant difference.

## Results

### DCLK1 promoted the EMT process through mediation of Notch1 signalling in GC cells

**Fig. 1:**
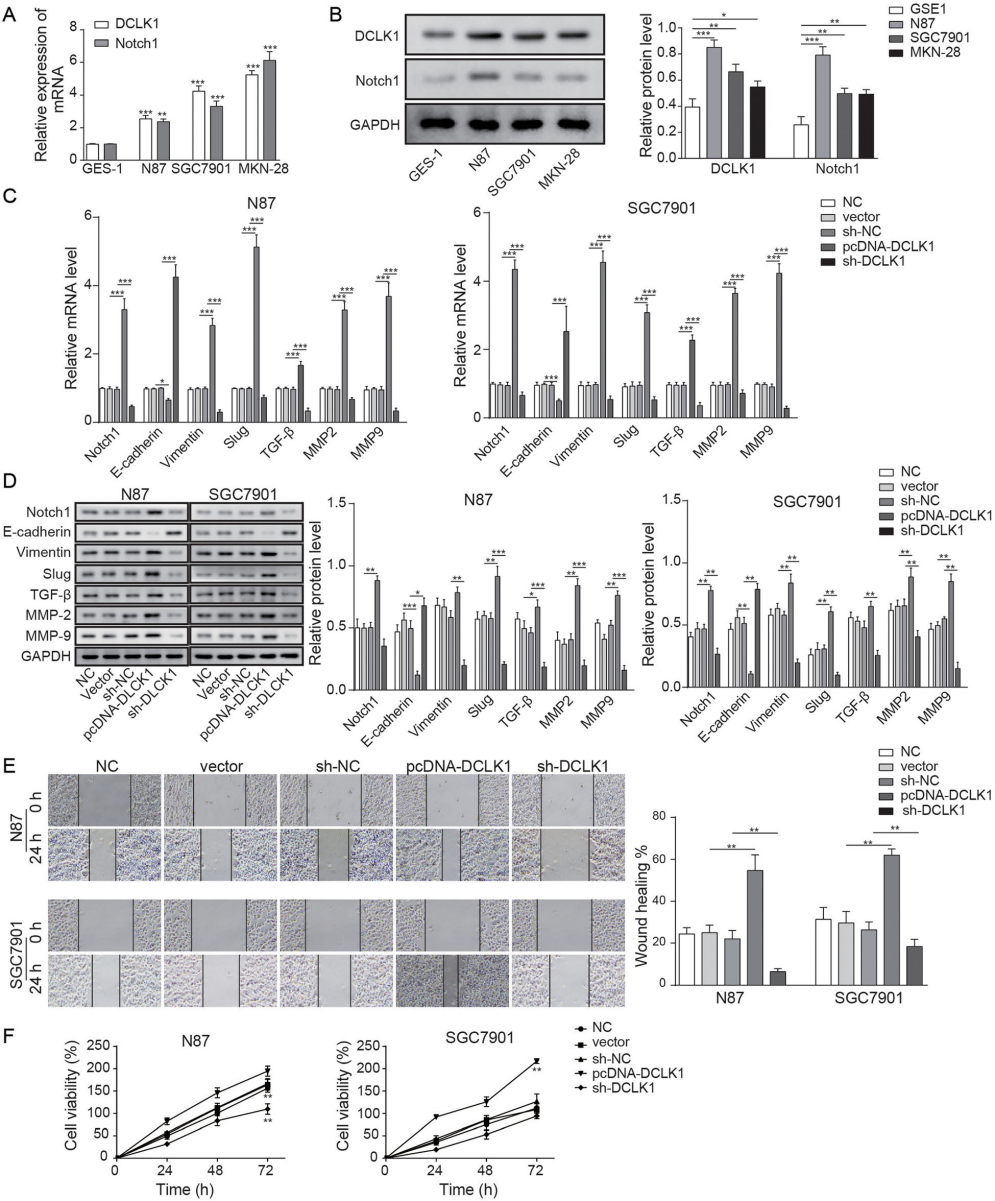
DCLK1 regulates the EMT process through modulating Notch1 in GC cells. **a**-**b** The relative expression of DCLK1 and Notch1 in human gastric mucosal cells (GES-1) and human GC cells (N87, SGC7901 and MKN-28) were detected by qRT-PCR (**a**) and Western blotting (**b**). **c**-**d** The relative expressions of Notch1 and EMT-related proteins were tested by qRT-PCR (C) and Western blotting (**d**). **e** The migration of N87 and SGC7901 cells transfected by overexpression or knockdown of DCLK1 was detected by Transwell assay. **f** The cell viability was detected by MTT assay after incubation for 24, 48 and 72 h. ^*^P < 0.05, ^**^P < 0.01, and ^***^P < 0.001 compared with the NC group

To investigate the role of DCLK1 in GC, the DCLK1 and Notch1 expression in human gastric cancer cell lines (N87, SGC7901 and MKN-28) and normal gastric mucosal cells (GES-1) was detected using qRT-PCR and Western blotting. In Fig. 1a, b, the DCLK1 or Notch1 expression was markedly enhanced in GC cell lines compared with normal gastric mucosal cells.

To further investigate the role of DCLK1 in the EMT process of GC, qRT-PCR and Western blotting were performed. As shown in Fig. 1c, d, the expression of Notch1, Slug, TGF-β, MMP2, MMP9 and Vimentin in N87 or SGC7901 cells transfected by the overexpression of DCLK1 was significantly upregulated compared with that in the pcDNA-vector group (vector), whereas knockdown of DCLK1 (sh-DCLK1) exhibited the opposite effects. In contrast, the expression of E-cadherin in GC cells was significantly decreased by overexpression of DCLK1 but upregulated in the presence of DCLK1 knockdown. On the other hand, the expression of EMT-related proteins in either vector (N87 or SGC7901 cells transfected by empty vector) or the sh-NC group was slightly affected compared with the NC group. Then, the wound healing and MTT assays were conducted to investigate the migration and proliferation of N87 or SGC7901 cells. The results in Fig. 1e indicated that cell migration in pcDNA-DCLK1 was significantly enhanced compared with vector (NC), which was inhibited by sh-DCLK1. Meanwhile, there was no significant change in cell migration in vector or sh-NC. Meanwhile, Fig. 1f suggested that the cell viability of pcDNA-DCLK1 was significantly increased compared with vector while DCLK1 knockdown significantly decreased the cell viability of GC. These results indicated that DCLK1 promotes the EMT process in GC cells via activation of the Notch1 signalling pathway.

### LncRNA SNHG1 mediated the DCLK1/Notch1 axis in GC cells

**Fig. 2:**
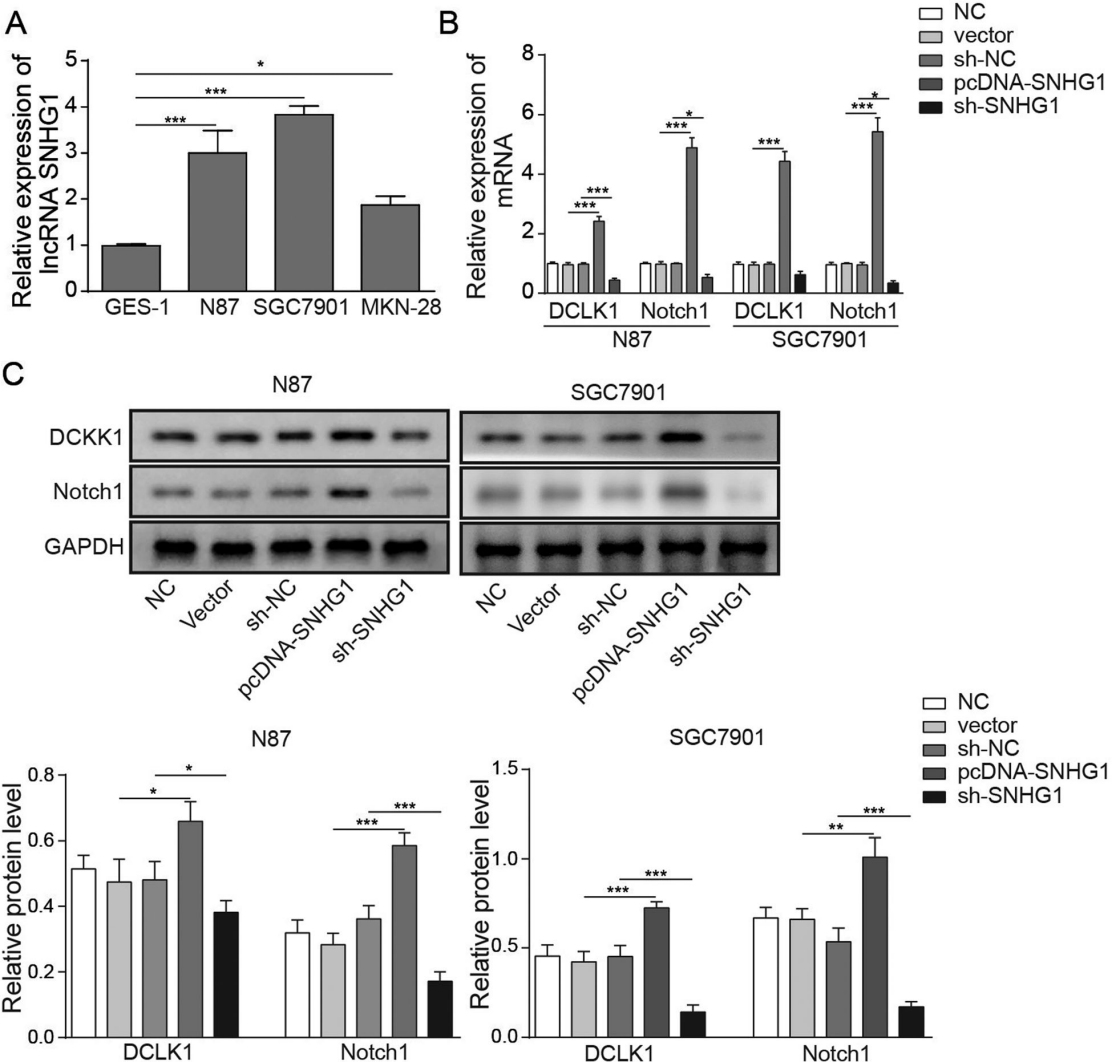
LncRNA SNHG1 mediates the DCLK1/Notch1 axis in GC cells. **a** The relative expressions of lncRNA SNHG1 in GES-1, N87, SGC7901 and MKN-28 cells were detected by qRT-PCR. **b**-**c** The relative expressions of DCLK1 and Notch1 in GC cells transfected by overexpression or knockdown of SNHG1 were measured by qRT-PCR (**b**) and Western blotting (**c**), respectively. ^*^P < 0.05, ^**^P < 0.01, and ^***^P < 0.001 compared with the NC group

Next, we aimed to confirm the role of lncRNA SNHG1 in GC; hence, the gene and protein expressions in GC cells were investigated using qRT-PCR and Western blotting, respectively. As indicated in Fig. 2a, the relative expression of SNHG1 in GC cell lines was significantly increased compared with normal gastric mucosal cells. Afterwards, to further confirm the role of lncRNA SNHG1 in the DCLK1/Notch1 axis during the progression of GC, the expressions of DCLK1 and Notch1 in N87 or SGC7901 cells were investigated. Figure 2b, c indicated that the expressions of DCLK1 or Notch1 were notably increased in SNHG1 overexpression compared with NC but were decreased in sh-SNHG1. These results indicated that lncRNA SNHG1 was differentially expressed and that it regulated the expression of DCLK1/Notch1 in GC cells.

### LncRNA SNHG1 affected EMT-related proteins through sponging miR-15b

**Fig. 3:**
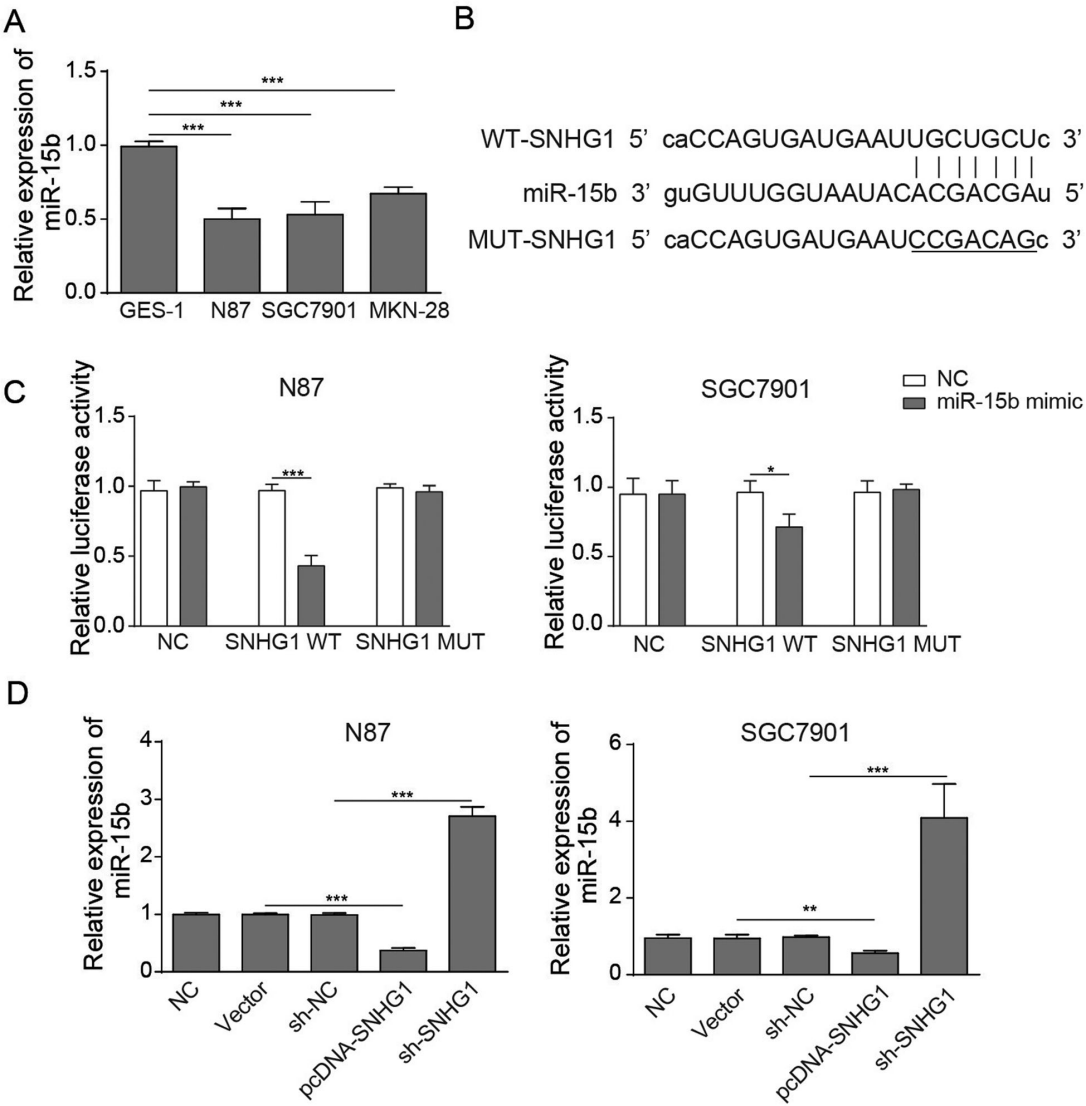
LncRNA SNHG1 affects EMT-related proteins through sponging miR-15b. **a** The relative expression of miR-15b in GES-1, N87, SGC7901 and MKN-28 cells was detected by qRT-PCR. **b** The lncRNA SNHG1 binding site to miR-15b was analysed using bioinformatics prediction software. **c** GC cells were co-transfected with either miR-15b mimic or NC mimics and pmirGLO Dual-Luciferase miRNA Target Expression Vector with WT or MUT 3′-UTR of SNHG1. Relative luciferase activity was determined by a dual-luciferase assay system. **d** The relative expressions of miR-15b in NC, vector, sh-NC, pcDNA SNHG1 and sh-SNHG1 were detected by qRT-PCR. ^*^P < 0.05, ^**^P < 0.01, and ^***^P < 0.001 compared with the NC group

To investigate the mechanism by which lncRNA SNHG1 affected the EMT process, the binding site of lncRNA SNHG1 and miR-15b was analysed by bioinformatics prediction software (Fig. 3b). The binding site was detected by dual-luciferase. The expression of miR-15b was detected by qRT-PCR, and Western blotting was used to detect EMT-related protein expression in GC cells. As demonstrated in Fig. 3a, the expression level of miR-15b in GC cell lines was significantly decreased compared with that in normal gastric mucosal cells (GES-1). Then, a dual-luciferase reporter assay was used to verify the relationship between miR-15b and SNHG1. The dual-luciferase reporter assay revealed that miR-15b might be the downstream target of SNHG1. In addition, qRT-PCR was used to illustrate the expression of miR-15b in GC cells transfected by SNHG1. Figure 3d indicates that miR-15b was clearly activated by knockdown of SNHG1 while it was inhibited by overexpression of SNHG1. These data indicate that lncRNA SNHG1 regulated the EMT process in GC by modulating the expression of miR-15b.

### MiR-15b mediated the expression of EMT-related proteins via targeting DCLK1

**Fig. 4:**
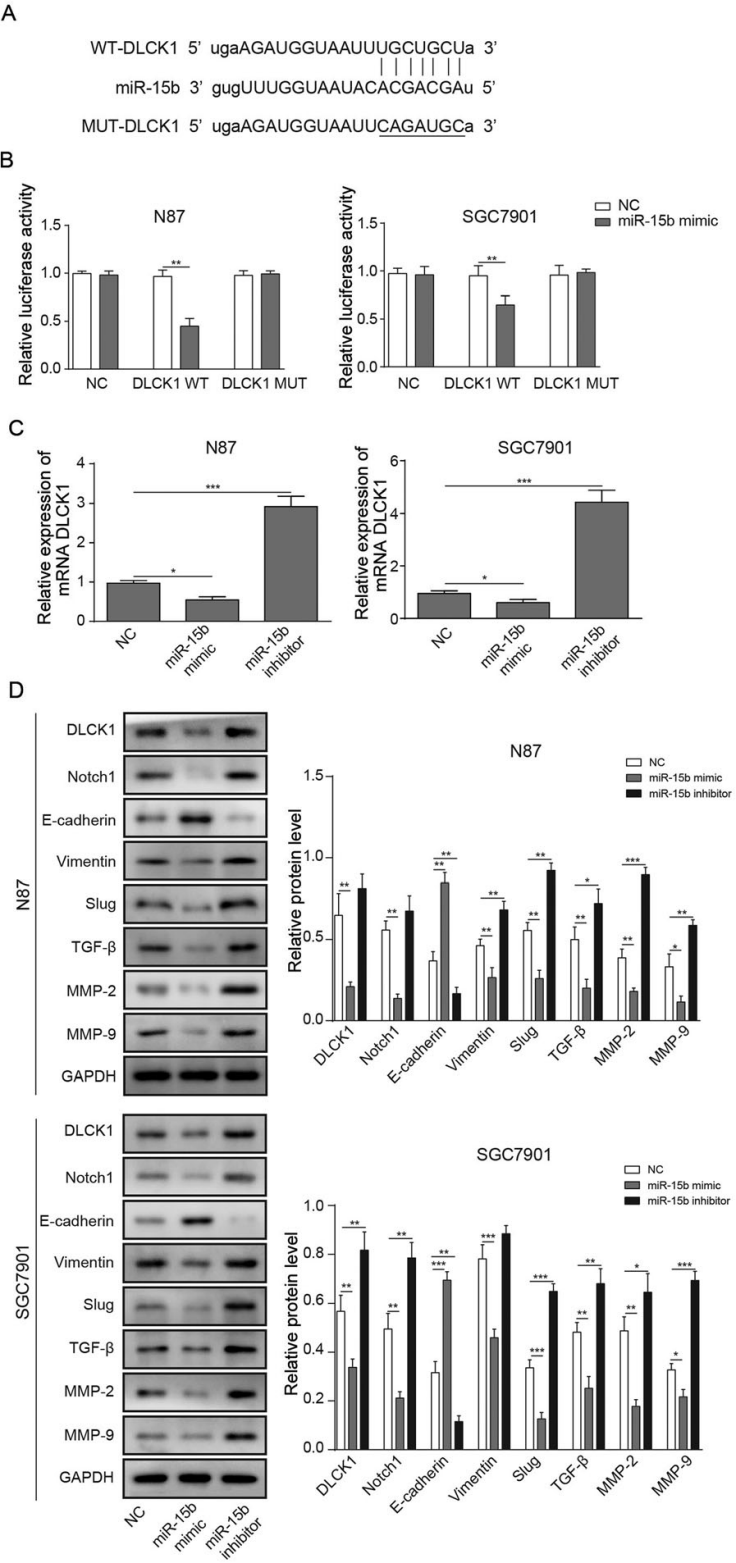
MiR-15b regulates EMT-related proteins via targeting DCLK1. **a** The binding site of miR-15b to DCLK1 was analysed using bioinformatics prediction software. **b** GC cells were co-transfected with either miR-15b mimic or NC mimic and pmirGLO Dual-Luciferase miRNA Target Expression Vector with WT or MUT 3′-UTR of DCLK1. Relative luciferase activity was determined by a dual-luciferase assay system. **c** The relative expressions of DCLK1 in NC, miR-15b mimics and miR-15b inhibitor were detected by qRT-PCR. **d** The expressions of DCLK1, Notch1 and EMT-related proteins in NC, miR-15b mimic and miR-15b inhibitor were detected by Western blotting. ^*^P < 0.05, ^**^P < 0.01, and ^***^P < 0.001 compared with the NC group

Then, we decided to investigate the mechanism by which miR-15b regulates EMT-related expression of GC; thus, bioinformatics prediction software was conducted to analyse the relationship between miR-15b and DCLK1 (Fig. 4a), double luciferase reporter assay was used to detect the binding site, qRT-PCR was used to investigate the expression of DCLK1, and Western blot detection was used to verify the qRT-PCR results and to detect the Notch1 signalling pathway and EMT-related proteins. As shown in Fig. 4b, the results indicate that DCLK1 is a direct target of miR-15b. The mRNA-related expression of DCLK1 in GC cells transfected with miR-15b inhibitor was significantly enhanced compared with the NC group but suppressed by miR-15b mimic (Fig. 4c). To verify the qRT-PCR result, protein expression in the GC cells was detected using Western blotting. The results indicate that the expressions of DCLK1, Notch1, TGF-β, Vimentin, MMP2, MMP9 and Slug in the GC cells transfected with miR-15b inhibitor were significantly higher compared with NC while miR-15b mimic exhibited the opposite functions. In contrast, the expression of E-cadherin in GC cells was notably decreased by downregulation of miR-15b but enhanced in the presence of miR-15b mimics (Fig. 4d). All of these data suggest that miR-15b can regulate the DCLK1/Notch1 axis in GC cells.

### LncRNA SNHG1 regulated the EMT process of GC via regulation of the DCLK1/Notch1/miR-15b axis

**Fig. 5:**
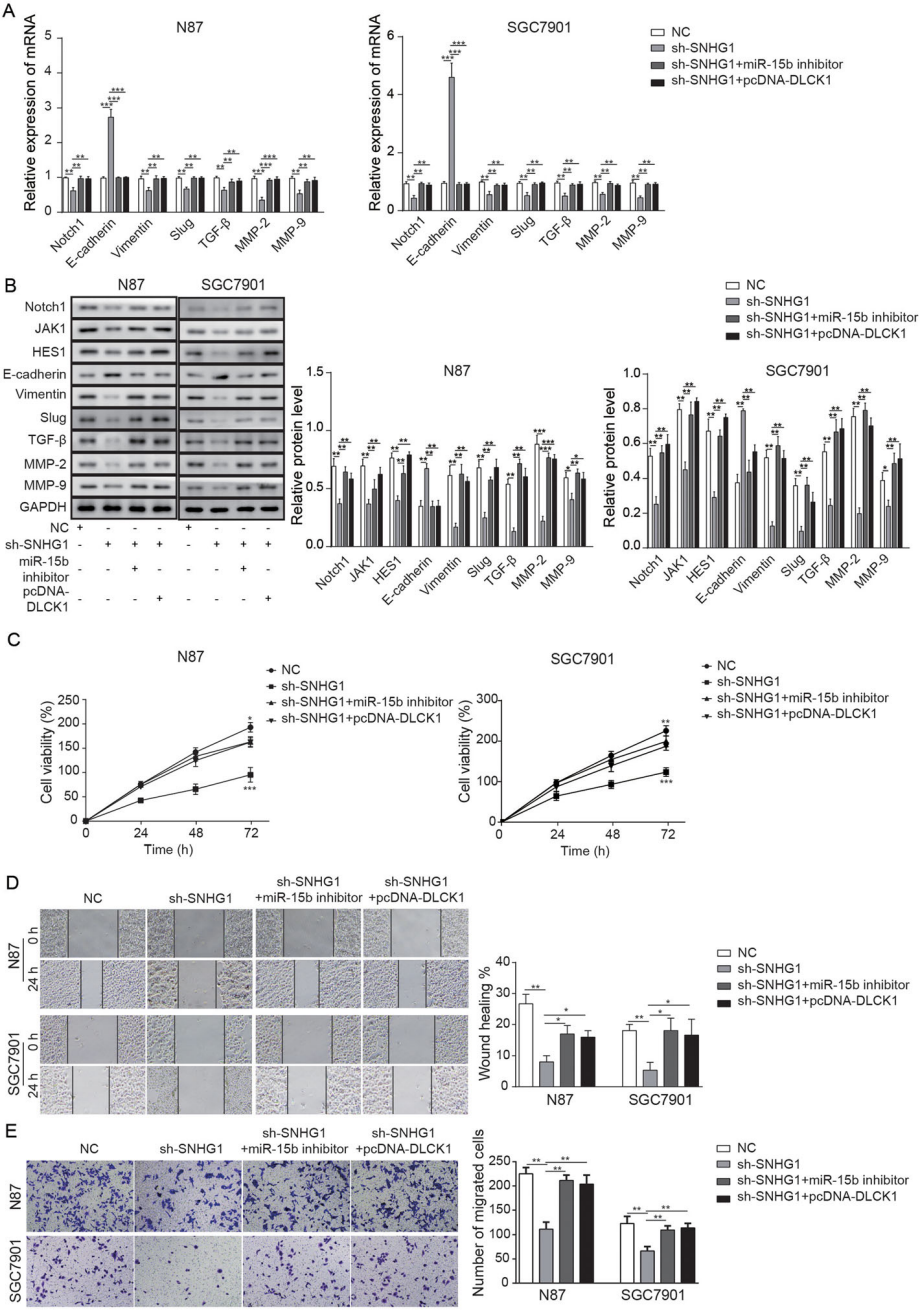
LncRNA SNHG1 promotes the EMT process in GC via modulation of the miR-15b/DCLK1/Notch1 axis. **a** The relative expressions of Notch1, E-cadherin, TGF-β, MMP2, MMP9, Slug and Vimentin in NC, sh-SNHG1, sh-SNHG1 + miR-15b inhibitor and sh-SNHG1 + pcDNA-DCLK1 were detected by qRT-PCR. **b** The expressions of Notch1 and EMT-related proteins in NC, sh-SNHG1, sh-SNHG1 + miR-15b inhibitor and sh-SNHG1 + pcDNA-DCLK1 were detected by Western blotting. **c** The cell viability value in NC, sh-SNHG1, sh-SNHG1 + miR-15b inhibitor and sh-SNHG1 + pcDNA-DCLK1 was tested after incubation for 24, 48 and 72 h using MTT assay. **d** The cell migration in NC, sh-SNHG1, sh-SNHG1 + miR-15b inhibitor and sh-SNHG1 + pcDNA-DCLK1 was tested by a wound healing assay. **e** The cell invasion in NC, sh-SNHG1, sh-SNHG1 + miR-15b inhibitor and sh-SNHG1 + pcDNA-DCLK1 was tested after by Transwell assay. ^*^P < 0.05, ^**^P < 0.01, and ^***^P < 0.001 compared with the NC group

To verify the results of previous studies, the expression of Notch1 and EMT-related protein in GC cells was detected by qRT-PCR and Western blotting. A Transwell assay was used to detect the invasive ability of GC cells. Figure 5a illustrates that the relative expression of Notch1, Slug and Vimentin were significantly decreased in GC cells transfected by SNHG1 knockdown (sh-SNHG1), which was reversed by miR-15b inhibitor or pcDNA-DCLK1. In contrast, the expression of E-cadherin in GC cells was notably increased in the presence of SNHG1 knockdown while miR-15b inhibitor or overexpression of DCLK1 significantly reversed the effect of SNHG1 knockdown. Additionally, in the Western blot detection, the expression of Notch1, MMP2, MMP9, Slug and Vimentin was significantly decreased in sh-SNHG1 while miR-15b inhibitor or overexpression of DCLK1 partially rescued the inhibitory effect of sh-SNHG1. However, the protein expression of E-cadherin was significantly inhibited by knockdown of SNHG1, which was significantly reversed by miR-15b inhibitor or DCLK1 overexpression (Fig. 5b). Then, we aimed to verify the role of lncRNA SNHG1 in the proliferation of GC cells. As illustrated in Fig. 5c, the cell viability in sh-SNHG1 was significantly suppressed, but it was significantly rescued by miR-15b inhibitor or pcDNA-DCLK1. Moreover, the cell migration was detected by wound healing. The results showed that cell migration was significantly downregulated by SNHG1 silencing, which was significantly increased by miR-15b inhibitor or pcDNA-DCLK1 (Fig. 5d). Finally, to detect the cell invasion ability, a Transwell assay was used. In Fig. 5e, the invasion of GC cells was significantly decreased by knockdown of SNHG1. Moreover, miR-15b inhibitor or pcDNA-DCLK1 enhanced the invasive effect of GC cells. Altogether, these results suggested that lncRNA SNHG1 regulates the effects of DCLK1/Notch1 on the EMT process and cell migration through mediating miR-15b in GC cells.

## Discussion

Many studies have suggested a relationship between tumour metastasis and the EMT process of cancer cells in GC. Zhang P. et al. found that tumour metastasis in GC can be affected by the progression of EMT in GC cells [16]. In addition, cell proliferation and migration can be promoted by an upregulated EMT process in GC [17–19]. These data may suggest that the EMT process plays a critical role in the progression of GC. However, he underlying mechanism of EMT remains largely unknown. Therefore, it is important to explore the role of EMT in GC. As expected, our current study confirmed the correlation between EMT and GC, indicating that EMT acts as a key regulator in the progression of GC.

DCLK1 has been regarded as a new potential cancer stem cell (CSC) marker in several types of cancer [20]. DCLK1 has been confirmed to play a promoting role in the progression of multiple malignant tumours [21–24]. Kalantari E. et al. found that DCLK1 was overexpressed in GC tissues but not significantly changed in adjacent tissues [6]. Hence, our finding further verified the function of DCLK1 in GC. Additionally, upregulated Notch1 signalling plays pivotal roles in the development of different types of human malignancies [25, 26]. Suppression of the Notch1 signalling pathway is considered to be a novel target for human cancer treatment [27, 28]. In this study, silencing of DCLK1 significantly inhibited the cell proliferation and migration of GC. In addition, our study found that Notch1 was significantly inactivated in the presence of DCLK1 knockdown, which was consistent with the previous study [29], indicating that DCLK1 may regulate tumour metastasis and the EMT process of GC via mediation of the Notch1 signalling pathway.

Studies have found that lncRNA is involved in many life activities such as the dose compensation effect, and it has become a hot topic in genetic research [30–32]. Many studies have reported that lncRNA is involved in regulating the progression of malignant tumours [33]. Mao Z. et al. found that lncRNA DANCR promotes tumour metastasis and cell invasion through suppression of lncRNA-LET in GC cells [34]. LncRNA SNHG1 involvement in the progression of multiple cancers has been confirmed [35, 36]. Meanwhile, Thin KZ et al. found that SNHG1 might act as an oncogene in GC [12]. In the present research, we found that SNHG1 silencing could induce cell growth inhibition of GC. This finding was consistent with the previous report that SNHG1 resulted in the cell proliferation of gastric cancer [37], confirming that SNHG1 is upregulated in the tumorigenesis of GC.

Additionally, the relationship between lncRNA and microRNA (miRNA) in progression of multiple diseases has been extensively studied. For instance, a previous study demonstrated that lnc00052 could regulate CALCOCO1 expression via regulating miR-574-5p in colorectal cancer (CRC) [38]. Moreover, some researchers have found that miRNA can be regulated by lncRNA to regulate the EMT process of cancer [39–41]. It has been previously reported that miR-15b could play a key role in human malignancies [42]. Moreover, Xia L et al. indicated that miR-15b could significantly suppress the progression of GC [43]. In the current study, we found that miR-15b can inhibit the expression of DCLK1/Notch1 in GC, whereas lncRNA SNHG1 regulates DCLK1/Notch1 by miR-15b. Based on these data, miR-15b can be considered to act as a suppressor during the progression of GC, and lncRNA SNHG1 could regulate the effects of DCLK1/Notch1 on the EMT process and cell migration through miR-15b modulation in GC.

Frankly speaking, this study did not include in vivo experiments or data on immunofluorescence staining due to insufficient funding. However, it still provides a new program for the treatment of GC.

## Conclusions

In summary, lncRNA SNHG1 can regulate the effects of DCLK1/Notch1 on the EMT process and cell migration in gastric cancer through miR-15b regulation, providing a novel potential target for GC treatment and also new hope for GC patients.

## Data Availability

All data generated or analysed during this study are included in this published article [and its supplementary information files].

## Abbreviations

GC: Gastric cancer
EMT: Epithelial-mesenchymal transition
lncRNA: Long non-coding RNA
DCLK1: Doublecortin-like kinase 1
SNHG1: Small nucleolar RNA host gene 1
miR-15b: microRNA-15b
